# Association between time to emergency neurosurgery and clinical outcomes for spontaneous hemorrhagic stroke: A nationwide observational study

**DOI:** 10.1371/journal.pone.0267856

**Published:** 2022-04-28

**Authors:** Ki Hong Kim, Young Sun Ro, Jeong Ho Park, Joo Jeong, Sang Do Shin, Sungwoo Moon

**Affiliations:** 1 Department of Emergency Medicine, Seoul National University Hospital, Seoul, Korea; 2 Laboratory of Emergency Medical Services, Seoul National University Hospital Biomedical Research Institute, Seoul, Korea; 3 National Emergency Medical Center, National Medical Center, Seoul, Korea; 4 Department of Emergency Medicine, Seoul National University Bundang Hospital, Gyeonggi, Korea; 5 Department of Emergency Medicine, Korea University Ansan Hospital, Gyeonggi, Korea; Chinese Academy of Medical Sciences and Peking Union Medical College, CHINA

## Abstract

**Objective:**

Spontaneous hemorrhagic stroke is a devastating disease with high mortality and grave neurological outcomes worldwide. This study aimed to evaluate the association between the elapsed time from emergency department (ED) visit to emergency neurosurgery and clinical outcomes in patients with spontaneous hemorrhagic stroke.

**Methods:**

A nationwide cross-sectional study was conducted using the nationwide emergency database in Korea. Spontaneous hemorrhagic stroke patients who received neurosurgery within 12 hours of ED visit between January 2018 and December 2019 were enrolled. The main exposure was time to neurosurgery and the primary outcome was in-hospital mortality. Multivariable logistic regression was conducted.

**Results:**

Among 2,602 study populations (incidence rate: 2.5 per 100,000 person-years, 15.8% of SAH, 78.6% of ICH, and 5.6% of mixed type), 525 (20.2%) patients received surgery in the ultra-early (0–2 hours) group, 1,093 (42.0%) in the early (2–4 hours) group, and 984 (37.8%) in the late (4–12 hours) group. The early group showed better survival outcomes than the ultra-early and late group (in-hospital mortality 22.2% vs. 26.5% and 26.1%, *p* = 0.06). Compared to the late group, adjusted OR (95% CI) for in-hospital mortality was 0.78 (0.63–0.96) for the early group, while there was no significant difference in the ultra-early group (0.90 (0.69–1.16)).

**Conclusions:**

Early neurosurgery within 2–4 hours of the ED visit was associated with favorable survival outcomes in patients with spontaneous hemorrhagic stroke.

## Introduction

Spontaneous hemorrhagic stroke is a devastating disease with high mortality and grave neurological outcomes worldwide [1]. As the aging population increases, the burden of the disease continues to increase due to the high incidence of the elderly and poor prognosis [2,3]. A recent study reported that the expected years of life lost for spontaneous hemorrhagic stroke was 6 to 9.7 years, and the expected lifetime cost was $86,327 [4]. Despite many efforts to improve clinical outcomes for patients with spontaneous hemorrhagic stroke, specific breakthrough treatments have not been developed [5].

The effects of time to vascular interventions on clinical outcomes have been well investigated in emergency cardio- and cerebro-vascular diseases such as acute myocardial infarction and ischemic stroke [6,7]. In terms of hemorrhagic stroke, early intervention is recommended to preserve the nervous system [8,9], reduce intracranial pressure (ICP) and improve neurological outcomes for subarachnoid hemorrhage (SAH) [10].

The elapsed time from emergency department (ED) visits to definitive care is associated with survival outcomes for patients with critical illnesses, and better outcomes are associated with treatments initiated in the hours immediately after diagnosis. Overall, early intervention is known to be associated with favorable clinical outcomes for patients with spontaneous hemorrhagic stroke who have undergone emergency surgery [8–11]. However, the gold standard of time to initiate surgery has not been well studied, and there are no emergency operation protocols to improve clinical outcomes and reduce complications for patients with spontaneous hemorrhagic stroke [12].

We hypothesized that a delay of operation reduces the chance of survival and increases complications, including reoperations, for patients with spontaneous hemorrhagic stroke. The purpose of this study was to evaluate the association between time elapsed from ED admission to surgery and clinical outcomes in patients with spontaneous hemorrhagic stroke who need emergency neurosurgery, and to determine the optimal cutoff time intervals for favorable survival outcomes.

## Methods

### Study design and data source

A nationwide cross-sectional study was conducted using the National Emergency Department Information System (NEDIS) database in Korea. The Ministry of Health and Welfare established the emergency medicine information network to measure emergency care qualities in 2003. The database collects data in real time from EDs nationwide, including demographics, treatments in the ED and hospital, and clinical outcomes. All information is automatically transferred from each hospital to a central government server following patients’ discharge from an ED or hospital. Inaccurate data is filtered by a data processing system. The NEDIS data is updated in real time by the National Emergency Medical Center and approved annually by National Statistics for data quality management [13,14].

### Study setting

Korea has approximately 50 million residents living in 17 provinces. The Ministry of Health and Welfare designated 3 levels of ED according to emergency medical resources and capacity (including facilities, equipment, and medical staff) and operated 38 Level 1 EDs, 125 Level 2 EDs, and 239 Level 3 EDs (a total of 402 EDs) in 2020. A Level 1 ED is designed to provide care for serious and severe emergency patients. Level 1 and Level 2 EDs are mostly assigned on-call neurosurgeons in the hospital, and Level 1 EDs have an emergency operating room in the ED. Hemorrhagic stroke is mostly diagnosed by computed tomography. Most hospitals follow standard protocols for management of hemorrhagic stroke including ICP monitoring, medical management, and determination of surgery [15,16].

The emergency medical services (EMS) system is a government-based system operated by the National Fire Agency. EMS providers perform basic to intermediate levels of prehospital care at the scene and transport suspected stroke patients to nearby Level 1 or Level 2 EDs, according to standard operating protocols. Interhospital transport is mainly performed by private EMS agencies that are regulated by the EMS Act.

### Study population

Patients who were diagnosed with spontaneous hemorrhagic stroke and received neurosurgery within 12 hours of admission to an ED between January 1, 2018, and December 31, 2019 were included. Patients with unknown information on outcomes and patients who received procedures delayed for more than 12 hours were excluded.

Patients with spontaneous hemorrhagic stroke were defined as patients who visited the ED with medical illnesses rather than injury and who were diagnosed with hemorrhagic stroke based on the International Statistical Classification of Diseases and Related Health Problems 10th Revision (ICD-10) code: I60 for SAH and I61 for ICH, with or without intraventricular hemorrhage. Patients diagnosed with subdural hemorrhage or epidural hemorrhage (ICD-10, I62) and those with traumatic brain injuries (ICD-10, S06) were not included.

Emergency neurosurgery was performed for spontaneous hemorrhagic stroke, including intraventricular burr-hole trephination, ventriculostomy, intraventricular hematoma evacuation, resection of cerebral arteriovenous malformation, craniotomy, and craniectomy [17]. Other vascular interventions, such as coil embolization, were not included.

### Exposure and outcome measurements

The exposure of interest was the time from ED visit to surgery. Information on the time of surgery in the NEDIS database was collected based on the time of skin incision. Restricted cubic spline analysis was performed to evaluate cutoff values for time intervals associated with survival outcomes.

The primary outcome was in-hospital mortality. The secondary outcome was reoperation defined as any neurosurgery during ED/hospital admission following the first emergency operation. Although it has been assumed that reoperation usually represents major rebleeding or complications, it is not possible to distinguish between emergency reoperation and planned definite operation after emergency procedure in the NEDIS database.

Information on age, sex, EMS use, transfer from another hospital, level of ED, date and time of ED visit (MN–8AM, 8AM–4PM, and 4PM–MN), mental status at triage (alert, verbal response, pain response, and unresponsive), diagnosis at ED and hospital discharge (ICD-10 code), time and type of surgery (burr-hole trephination and other neurosurgery), disposition of ED and hospital, and in-hospital mortality were collected and analyzed.

### Statistical analysis

For the study population, age- and sex-specific incidence rates per 100,000 person-years were calculated by subtype of hemorrhagic stroke using the 2019 midyear census population. Demographic and clinical findings with outcomes according to main exposure were described. Statistical differences between study groups were analyzed using the Wilcoxon rank sum test for continuous variables and the chi-square test for categorical variables.

Multivariable logistic regression analysis was conducted to evaluate the association between study groups and outcomes. Adjusted odds ratios (ORs) with 95% confidence intervals (CIs) were calculated. Potential confounders were selected, including age group (<50, 50–69, and over 70 years old), sex, EMS use, transfer from another hospital, level of ED (Level 1 and others), mental status at ED triage, type of hemorrhagic stroke (SAH, ICH, and mixed), and combined intraventricular hemorrhage.

Sensitivity analyses were performed to determine the optimal cutoff point for the elapsed time from the ED visit to surgical decompression, which is associated with survival outcomes. To enhance clinical utility, the elapsed time variable was dichotomized at each cutoff value in this analysis.

All statistical analyses were performed with SAS, version 9.4 (SAS, Cary, SC, USA), Microsoft Excel software (Microsoft Corp. 2020) and R, version 3.5 (R Foundation for Statistical Computing, Vienna, Austria) with the Hmisc package.

### Ethics statement

This study complies with the Declaration of Helsinki. Its protocol was approved by the Institutional Review Board (IRB No. 2012-104-1183), and the requirement for informed consent was waived due to the retrospective nature of this study.

## Results

**Fig 1:**
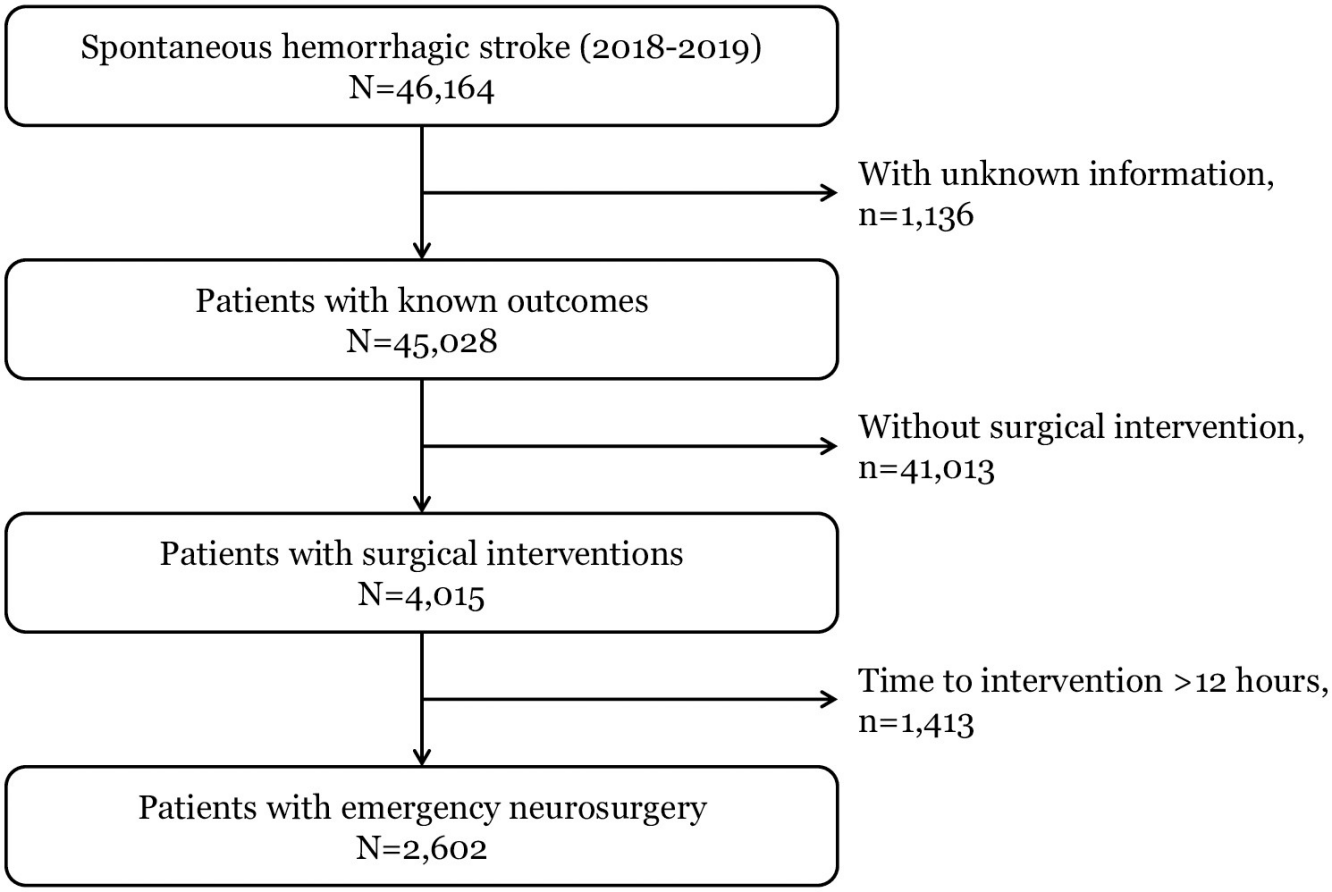
Study population flow.

Among 46,164 patients (incidence rate: 46.2 per 100,000 person-years) who visited the ED and were diagnosed with spontaneous hemorrhagic stroke during the study period, a total of 2,602 patients (incidence rate: 2.5 per 100,000 person-years) were included in the final analysis after excluding 1,136 patients with unknown information on outcomes, 41,013 patients who did not receive procedures and 1,413 patients who received surgery delayed for more than 12 hours (Fig 1).

**Fig 2:**
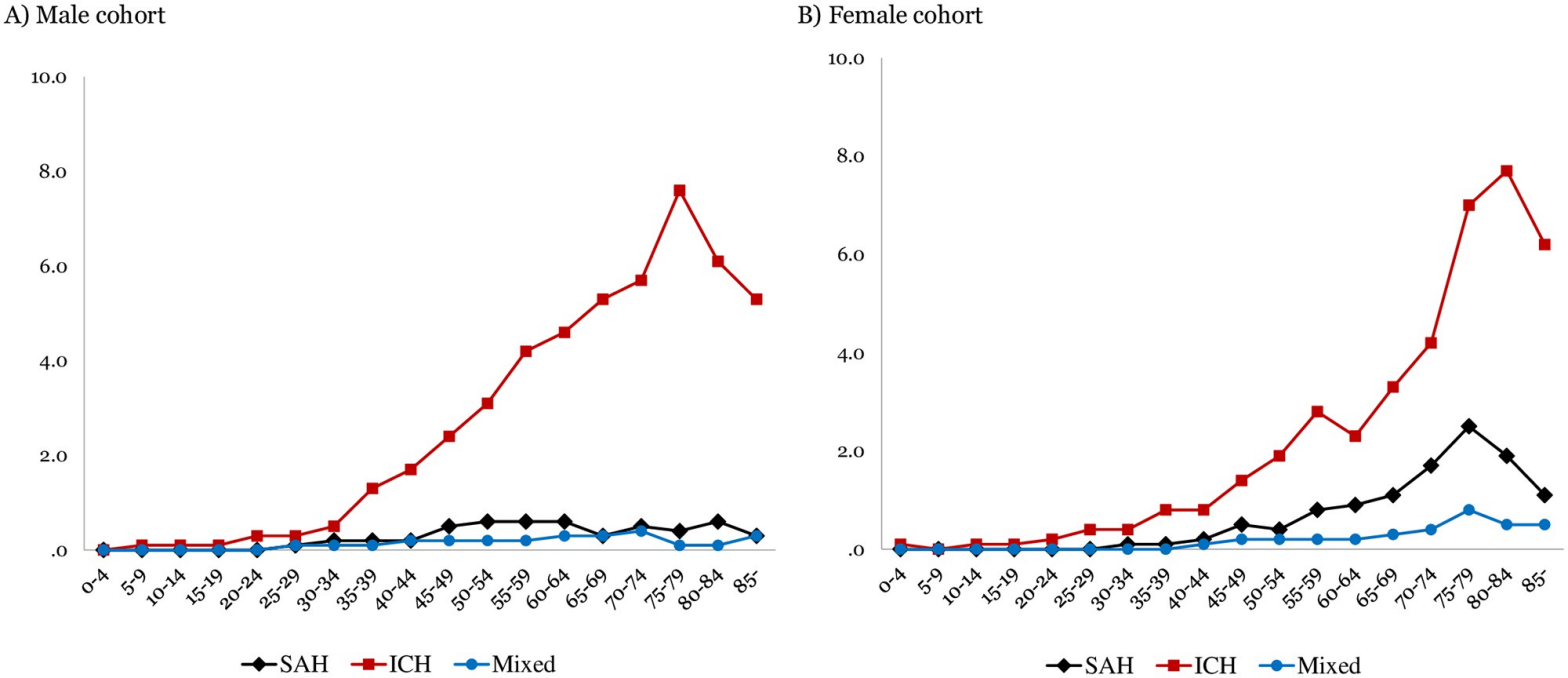
Age- and sex-specific incidence rates of spontaneous hemorrhagic stroke with emergency neurosurgery. SAH, subarachnoid hemorrhage; ICH, intracerebral hemorrhage.

Age- and sex-specific incidence rates per 100,000 person-years are demonstrated by subtype of hemorrhagic stroke in Fig 2.

**Fig 3:**
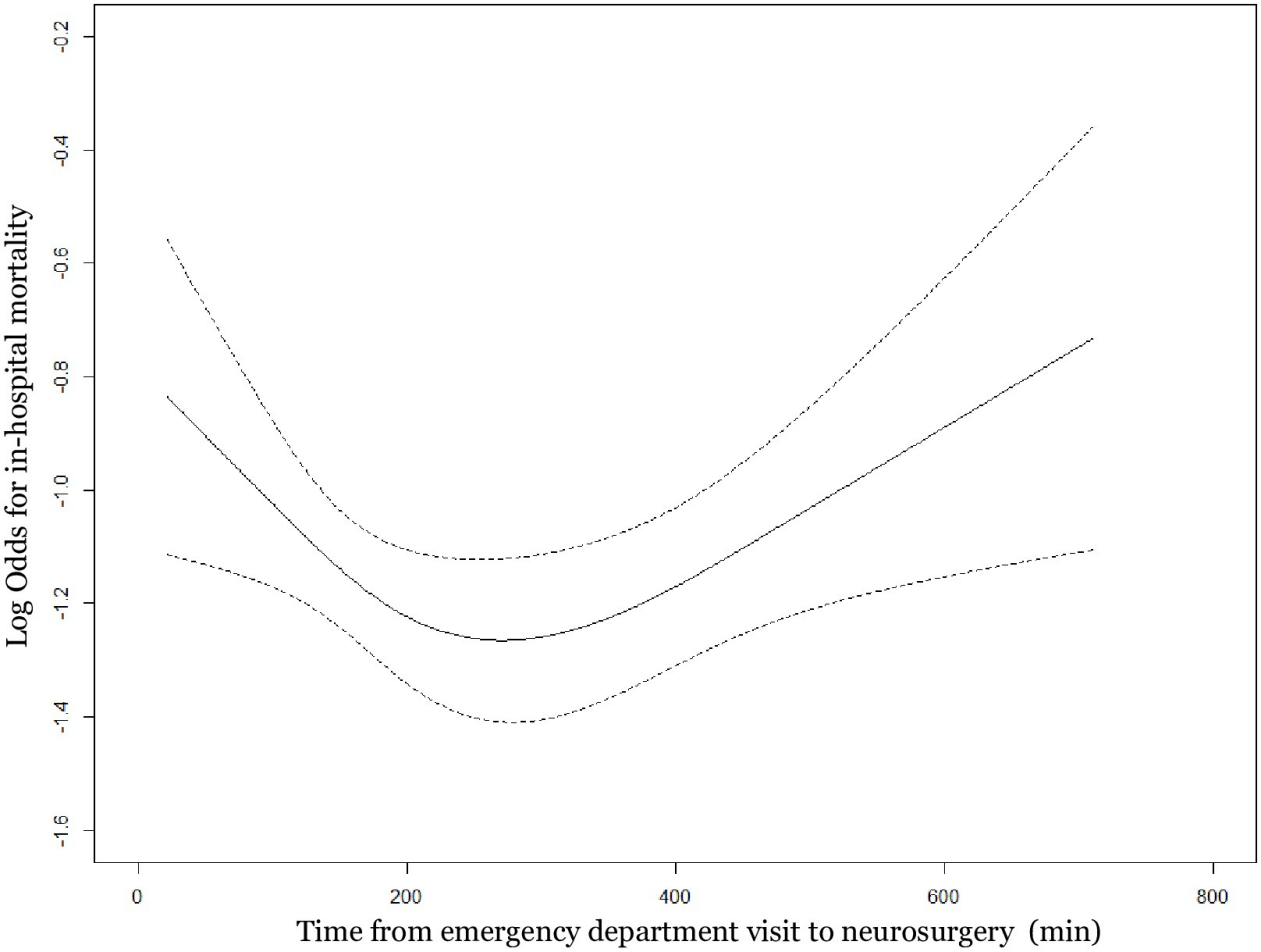
Restricted cubic spline graph of time from emergency department visit to neurosurgery and in-hospital mortality.

The restricted cubic spline graph between the time from ED admission to neurosurgery and in-hospital mortality for the study population is shown in Fig 3. Based on the U-shaped graph, 2 hours and 4 hours were considered cutoff values.

Demographics and clinical characteristics were compared between the ultra-early (0–2 hours), early (2–4 hours), and late (4–12 hours) groups; 525 (20.2%) patients received surgery in the ultra-early group, 1,093 (42.0%) in the early group, and 984 (37.8%) in the late group. In-hospital mortalities were 24.6% among the study population, 26.5% in the ultra-early group, 22.2% in the early group, and 26.1% in the late group (p-value, 0.06) (Table 1).

**Table 1:** Demographics of study population according to time from emergency department visit to emergency neurosurgery.

	Time from ED visit to neurosurgery				
	Total	Ultra-early (0–2 hours)	Early (2–4 hours)	Late (4–12 hours)	p-value
	N (%)	N (%)	N (%)	N (%)	
Total	2,602	525 (20.2)	1,093 (42.0)	984 (37.8)	
Age, year, median (IQR)	61 (51–73)	61 (51–71)	61 (51–74)	61 (52–73)	0.71
0–49	562 (21.6)	116 (22.1)	252 (23.1)	194 (19.7)	0.48
50–59	649 (24.9)	134 (25.5)	260 (23.8)	255 (25.9)	
60–69	568 (21.8)	120 (22.9)	226 (20.7)	222 (22.6)	
70–79	538 (20.7)	106 (20.2)	224 (20.5)	208 (21.1)	
80–120	285 (11.0)	49 (9.3)	131 (12.0)	105 (10.7)	
Sex, male	1,341 (51.5)	268 (51.0)	552 (50.5)	521 (52.9)	0.52
EMS use	1,670 (64.2)	330 (62.9)	703 (64.3)	637 (64.7)	<0.01
Transferred from other hospital	789 (30.3)	172 (32.8)	339 (31.0)	278 (28.3)	0.16
Level 1 ED	1,185 (45.5)	251 (47.8)	513 (46.9)	421 (42.8)	0.08
Time of ED visit					<0.01
MN–8AM	482 (18.5)	84 (16.0)	182 (16.7)	216 (22.0)	
8AM–4PM	1,138 (43.7)	220 (41.9)	462 (42.3)	456 (46.3)	
4PM–MN	982 (37.7)	221 (42.1)	449 (41.1)	312 (31.7)	
Mental status at triage					<0.01
Alert	627 (24.1)	82 (15.6)	256 (23.4)	289 (29.4)	
Verbal response	500 (19.2)	85 (16.2)	216 (19.8)	199 (20.2)	
Pain response	1,114 (42.8)	257 (49.0)	483 (44.2)	374 (38.0)	
Unresponsive	361 (13.9)	101 (19.2)	138 (12.6)	122 (12.4)	
Hemorrhagic stroke					<0.01
SAH	411 (15.8)	45 (8.6)	155 (14.2)	211 (21.4)	
ICH	2,044 (78.6)	460 (87.6)	879 (80.4)	705 (71.6)	
Mixed	147 (5.6)	20 (3.8)	59 (5.4)	68 (6.9)	
Combined IVH	701 (26.9)	187 (35.6)	309 (28.3)	205 (20.8)	<0.01
Type of neurosurgery					<0.01
Burr-hole, in ED	226 (8.7)	83 (15.8)	111 (10.2)	32 (3.3)	
Burr-hole, in operation room	1,250 (48.0)	248 (47.2)	556 (50.9)	446 (45.3)	
Other neurosurgery	1,126 (43.3)	194 (37.0)	426 (39.0)	506 (51.4)	
ED disposition					<0.01
ICU admission	500 (19.2)	94 (17.9)	141 (12.9)	265 (26.9)	
Operation room	2,048 (78.7)	423 (80.6)	923 (84.4)	702 (71.3)	
Reoperation	332 (12.8)	61 (11.6)	136 (12.4)	135 (13.7)	0.47
In-hospital mortality	639 (24.6)	139 (26.5)	243 (22.2)	257 (26.1)	0.06

ED, emergency department; IQR, interquartile range; EMS, emergency medical services; SAH, subarachnoid hemorrhage; ICH, intracerebral hemorrhage; IVH, intraventricular hemorrhage; ICU, intensive care unit.

By subtype of spontaneous hemorrhagic stroke, 10.9% of SAH, 22.5% of ICH, and 13.6% of mixed-type hemorrhage patients received neurosurgery in the ultra-early interval group. The in-hospital mortality rates were 128 (31.1%) in the SAH, 457 (22.4%) in the ICH, and 54 (36.7%) in the mixed hemorrhage groups (Table 2).

**Table 2:** Demographics of study population by subtype of spontaneous hemorrhagic stroke.

	Hemorrhagic stroke		
	Subarachnoid hemorrhage	Intracerebral hemorrhage	Mixed
	N (%)	N (%)	N (%)
Total	411	2,044	147
Time to neurosurgery			
Ultra-early (0–2 hours)	45 (10.9)	460 (22.5)	20 (13.6)
Early (2–4 hours)	155 (37.7)	879 (43.0)	59 (40.1)
Late (4–12 hours)	211 (51.3)	705 (34.5)	68 (46.3)
Age, year, median (IQR)	60 (51–73)	61 (52–73)	60 (49–72)
0–49	86 (20.9)	437 (21.4)	39 (26.5)
50–59	105 (25.5)	512 (25.0)	32 (21.8)
60–69	87 (21.2)	450 (22.0)	31 (21.1)
70–79	92 (22.4)	415 (20.3)	31 (21.1)
80–120	41 (10.0)	230 (11.3)	14 (9.5)
Sex, male	141 (34.3)	1,134 (55.5)	66 (44.9)
EMS use	247 (60.1)	1,317 (64.4)	106 (72.1)
Transferred from other hospital	144 (35.0)	606 (29.6)	39 (26.5)
Level 1 ED	222 (54.0)	892 (43.6)	71 (48.3)
Time of ED visit			
MN–8AM	92 (22.4)	366 (17.9)	24 (16.3)
8AM–4PM	171 (41.6)	908 (44.4)	59 (40.1)
4PM–MN	148 (36.0)	770 (37.7)	64 (43.5)
Mental status at triage			
Alert	107 (26.0)	503 (24.6)	17 (11.6)
Verbal response	67 (16.3)	419 (20.5)	14 (9.5)
Pain response	153 (37.2)	879 (43.0)	82 (55.8)
Unresponsive	84 (20.4)	243 (11.9)	34 (23.1)
Combined IVH	0 (0.0)	610 (29.8)	91 (61.9)
Type of neurosurgery			
Burr-hole, in ED	26 (6.3)	190 (9.3)	10 (6.8)
Burr-hole, in operation room	240 (58.4)	941 (46.0)	69 (46.9)
Other neurosurgery	145 (35.3)	913 (44.7)	68 (46.3)
ED disposition			
ICU admission	67 (16.3)	417 (20.4)	16 (10.9)
Operation room	335 (81.5)	1,583 (77.4)	130 (88.4)
Reoperation	73 (17.8)	237 (11.6)	22 (15.0)
In-hospital mortality	128 (31.1)	457 (22.4)	54 (36.7)

IQR, interquartile range; ED, emergency department; IVH, intraventricular hemorrhage; EMS, emergency medical services; ICU, intensive care unit.

Among emergency neurosurgery, intraventricular burr-hole trephination was the most common type of surgery (57.9% for SAH and 52.4% for ICH), followed by intraventricular hematoma evacuation (35.0% for ICH) and craniotomy (26.3% for SAH) (Table 3).

**Table 3:** Emergency neurosurgery by subtype of spontaneous hemorrhagic stroke.

	Subarachnoid hemorrhage			Intracerebral hemorrhage			Mixed		
	Total	Reoperation	In-hospital mortality	Total	Reoperation	In-hospital mortality	Total	Reoperation	In-hospital mortality
	N	N (%)	N (%)	N	N (%)	N (%)	N	N (%)	N (%)
Total	411	73 (17.8)	128 (31.1)	2044	237 (11.6)	457 (22.4)	147	22 (15.0)	54 (36.7)
Intraventricular burr-hole trephination	238	34 (14.3)	64 (26.9)	1071	85 (7.9)	232 (21.7)	16	3 (18.8)	6 (37.5)
Ventriculostomy	6	2 (33.3)	2 (33.3)	4	1 (25.0)	1 (25.0)	76	10 (13.2)	30 (39.5)
Intraventricular hematoma evacuation	52	15 (28.8)	11 (21.2)	715	82 (11.5)	159 (22.2)	51	8 (15.7)	18 (35.3)
Resection of arteriovenous malformation	1	0 (0.0)	1 (100.0)	28	5 (17.9)	2 (7.1)	4	1 (25.0)	0 (0.0)
Craniotomy	108	21 (19.4)	47 (43.5)	215	63 (29.3)	59 (27.4)	0	-	-
Craniectomy	6	1 (16.7)	3 (50.0)	11	1 (9.1)	4 (36.4)	0	-	-

In multivariable logistic regression analysis, the early group showed better survival outcomes than the late group (adjusted OR (95% CI) for in-hospital mortality, 0.78 (0.63–0.96)), while there was no significant difference in the ultra-early group (adjusted OR (95% CI), 0.90 (0.69–1.16)) in Model 3. For reoperation, the adjusted ORs (95% CIs) were 0.92 (0.66–1.29) in the ultra-early group and 0.96 (0.74–1.24) in the early group (Table 4).

**Table 4:** Multivariable logistic regression analysis by time from emergency department visit to emergency neurosurgery.

	Model 1	Model 2	Model 3
	Adjusted OR (95% CI)	Adjusted OR (95% CI)	Adjusted OR (95% CI)
In-hospital mortality			
Ultra-early (0–2 hours)	1.03 (0.81–1.31)	0.89 (0.69–1.14)	0.90 (0.69–1.16)
Early (2–4 hours)	0.81 (0.67–1.00)	0.77 (0.62–0.95)	0.78 (0.63–0.96)
Late (4–12 hours)	1.00	1.00	1.00
Reoperation			
Ultra-early (0–2 hours)	0.83 (0.60–1.14)	0.92 (0.66–1.28)	0.92 (0.66–1.29)
Early (2–4 hours)	0.90 (0.70–1.17)	0.96 (0.74–1.24)	0.96 (0.74–1.24)
Late (4–12 hours)	1.00	1.00	1.00

OR, odds ratio; CI, confidence interval.

* Model 1: Adjusted for age, and sex.

* Model 2: Adjusted for variables in Model 1, mental status at triage, combined intraventricular hemorrhage, and type of hemorrhagic stroke.

* Model 3: Adjusted for variables in Model 2, emergency medical services use, transferred from other hospital, and level of emergency department.

To determine the optimal binary cutoff point for elapsed time from ED visit to emergency neurosurgery, four cutoffs at 30-minute intervals were selected considering the distribution of the study population (4, 4.5, 5, and 5.5 hours). For each cutoff, a multivariable logistic regression model was conducted. The lowest estimate in adjusted OR was observed in the 4.5-hour cutoff model (adjusted OR (95% CI), 0.80 (0.66–0.98)), and no significant estimate was found for reoperation in any time cutoffs (Table 5).

**Table 5:** Sensitivity analysis for cutoffs of time from emergency department visit to emergency neurosurgery.

	In-hospital mortality		Reoperation	
	n/N (%)	AOR (95% CI)	n/N (%)	AOR (95% CI)
Cut-off, 4 hours				
Early (0–4 hours)	382/1618 (23.6)	0.82 (0.67–0.99)	197/1618 (12.2)	0.95 (0.75–1.21)
Late (4–12 hours)	257/984 (26.1)	1.00	135/984 (13.7)	1.00
Cut-off, 4.5 hours				
Early (0–4.5 hours)	414/1748 (23.7)	0.80 (0.66–0.98)	214/1748 (12.2)	0.94 (0.74–1.21)
Late (4.5–12 hours)	225/854 (26.3)	1.00	118/854 (13.8)	1.00
Cut-off, 5 hours				
Early (0–5 hours)	453/1884 (24.0)	0.84 (0.68–1.04)	231/1884 (12.3)	0.92 (0.71–1.19)
Late (5–12 hours)	186/718 (25.9)	1.00	101/718 (14.1)	1.00
Cut-off, 5.5 hours				
Early (0–5.5 hours)	486/2020 (24.1)	0.80 (0.64–1.00)	248/2020 (12.3)	0.90 (0.68–1.18)
Late (5.5–12 hours)	153/582 (26.3)	1.00	84/582 (14.4)	1.00

ED, emergency department; AOR, adjusted odds ratio; CI, confidence interval.

Adjusted odd ratios were calculated adjusting for age, sex, emergency medical services use, transferred from other hospital, level of emergency department, mental status at triage, combined intraventricular hemorrhage, and type of hemorrhagic stroke.

## Discussion

Using a nationwide emergency patient database, this study demonstrated the effects of the elapsed time interval from ED admission to neurosurgery on clinical outcomes in patients with spontaneous nontraumatic hemorrhagic stroke who need emergency neurosurgery. A significant association was found between early surgery within 2–4 hours of the ED visit and favorable survival outcomes in patients with spontaneous hemorrhagic stroke (adjusted OR (95% CI), 0.78 (0.63–0.96) for in-hospital mortality). However, there was no association between the elapsed time intervals and reoperations after emergency neurosurgery. In sensitivity analyses, 4.5 hours from ED admission to surgery has been demonstrated as the optimal binary cutoff time interval to enhance survival outcomes. These results suggest that it is important to establish the gold standard of emergency neurosurgery and to develop standard protocols for EDs, such as critical pathways for patients with spontaneous hemorrhagic stroke, to maximize the survival gain and minimize complications.

Emergency surgical decompression of elevated ICP is important to prevent the acceleration of brain edema, reduce mortality and length of stay in the ICU, and improve functional recovery [18–21]. It has been suggested that susceptible patients should be treated as early as possible, as cerebral edema compresses adjacent tissues, causing secondary damage. Early interventions affect clinical outcomes for patients with time-sensitive emergency conditions. In patients with ST-segment elevation acute myocardial infarction, the elapsed time interval from the ED visit to coronary intervention is known to be associated with postevent ventricular function and short-term and long-term mortalities [22,23]. Therefore, providing coronary intervention to those patients within 90 minutes of ED visits is recommended as a standard worldwide protocol [24]. Similarly, for ischemic stroke, vascular interventions within the golden time period—defined as 4.5 hours from symptom onset—are associated with favorable neurologic recovery [25]. In this study, patients with spontaneous hemorrhagic stroke who needed emergency neurosurgery experienced better outcomes when early interventions began within 4.5 hours of their arrival at the ED. These results suggest that multistrategic improvements in the diagnosis and treatment process for patients with hemorrhagic stroke are needed to improve survival outcomes.

In this study, there was a U-shaped relationship between the time of ED arrival to neurosurgery and in-hospital mortality in the restricted cubic spline graph. The ultra-early group was not associated with survival benefits from neurosurgery compared to the late group. This phenomenon is commonly found in critically ill patients who need emergency interventions [26–28]. Healthcare professionals may tend to perform early life-saving procedures for severely ill patients. In this study, the proportion of emergency burr-hole trephination in the ED was higher in the ultra-early group than in other groups, and the proportions of patients with altered mental status and cases who were transferred from other hospitals were also larger in that group. These factors may contribute to the relatively poor survival outcomes of the ultra-early group, and the survival benefits found in the early group were absent in the ultra-early group.

Determining the indications for emergency neurosurgery in patients with spontaneous hemorrhagic stroke and establishing the gold standard for timely surgery are reasonable for improving the clinical outcomes of patients facing a disease with such high mortality. There is a strong need to develop standard protocols for EDs in detail, such as critical pathways for patients with spontaneous hemorrhagic stroke who need surgical decompression for elevated ICP, to maximize the survival gain and minimize complications that may worsen rapidly in the early phase of disease progression.

### Limitations

This study has several limitations. First, this study is a nationwide retrospective observational study. Potential biases could have affected the study results, and the generalizability of these data is limited. This is the inherent methodological limitation of observational studies. Second, information on the symptom onset, comorbidities, medical treatment, and disease severity of hemorrhagic stroke, including anatomical location of hemorrhage, amount of bleeding, estimated ICP, and main reason of neurosurgery are not collected in the NEDIS database. Although we limited the study population to patients who received neurosurgery within 12 hours of arrival in an ED, outcomes are limited to the in-depth interpretation of our results. Neurological or functional outcomes, such as modified Rankin Scale score, were also not be collected. In addition, since it is difficult to distinguish between emergency reoperation and planned definitive neurosurgery after emergency procedure, caution should be taken when interpreting these results given this significant limitation. Third, the time of neurosurgery is not the definite time of ICP reduction but the time of incision on the skin. Time to ICP reduction could affect the result but was unavailable in analysis.

## Conclusions

Early surgery was associated with favorable survival outcomes in patients with spontaneous hemorrhagic stroke who needed emergency neurosurgery; however, there was no association between the elapsed time intervals and reoperations after emergency neurosurgery. Determining surgical indications and developing standard protocols for those patients are needed to improve survival outcomes for patients facing this fatal disease.
